# Recombinant Human Endostatin Suppressed the Biological Behavior of Human Umbilical Vein Endothelial Cells Under Hypoxic and Hypoxic/Starvation Conditions In Vitro

**DOI:** 10.1155/ancp/3475731

**Published:** 2025-03-30

**Authors:** Yongsheng Jia, Cuicui Zhang, Jimin Zhao, Chuanxiang Hu, Xiaoyong Yang, Yan Zhang

**Affiliations:** ^1^Thyroid and Neck Department, Tianjin Medical University Cancer Institute and Hospital, Tianjin 300060, China; ^2^National Clinical Research Center for Cancer, Tianjin 300060, China; ^3^Key Laboratory of Cancer Prevention and Therapy, Tianjin 300060, China; ^4^Department of Thoracic Oncology, Tianjin Medical University Cancer Institute and Hospital, Tianjin 300060, China; ^5^Tianjin's Clinical Research Center for Cancer, Tianjin 300060, China

**Keywords:** biological behavior, HUVEC, hypoxia, hypoxia/starvation, normoxia, rh-endostatin

## Abstract

Recombinant human endostatin (rh-endostatin) has been shown to act as an inhibitor of angiogenesis. Previous studies have indicated that rh-endostatin combined with chemotherapy can improve the objective response rate (ORR), time to progression (TTP), and clinical benefit rate (CBR) without increasing toxicity. However, this function has seldom been reported in normal cells. The aim of our study was to explore the effect of rh-endostatin on the biological behavior of human umbilical vein endothelial cells (HUVECs) under different conditions in vitro. Confluent HUVECs were cultured under normoxic, hypoxic, or hypoxic/starvation (H/S) conditions and then treated with rh-endostatin. An MTT assay was used to assess cell proliferation, and HUVEC tube formation and migration were assessed via a cell tubule formation assay and a migration assay. The expression of endoglin (CD105) was assessed by flow cytometry (FCM). Rh-endostatin inhibited the proliferation, migration, and tube formation of HUVECs under normoxic, hypoxic, and H/S conditions. Compared with that in the normoxia group, the expression of CD105 was not different in the hypoxia 24 h group, but in the starvation and hypoxia/starvation groups, the expression of CD105 was upregulated. Rh-endostatin downregulated the expression of CD105 under all the study conditions. Here we found rh-endostatin suppressed the biological behavior of HUVECs under hypoxic and H/S conditions. As the concentration increased, the effect of rh-endostatin on the biological behavior of HUVECs was not greatly enhanced. Rh-endostatin did not promote malignant biological behavior or CD105 expression. Since CD105 may induce endothelial-to-mesenchymal transition in HUVECs, we hypothesized that rh-endostatin may inhibit the malignant biological behavior of HUVECs under hypoxic conditions in vitro.

## 1. Introduction

Angiogenesis plays a fundamental and critical role in solid-tumor pathogenesis, growth, invasion, and metastasis [1]. The development of malignancies involves multiple crucial steps, which mainly include vascular endothelial cell activation for proliferation and migration; homing to the neoplastic focus; and adhering to channels for vessel formation [2]. Tumor tissue can promote angiogenesis through signal release and complex transmission; new vessels can increase the supply of oxygen and nutrients, which can maintain hypermetabolism and prevent the accumulation of metabolites. In addition, tumor cells can enter the circulatory system through new blood vessels, causing distant metastases [3, 4]. However, antiangiogenic drugs can inhibit tumors by promoting the degradation of tumor blood vessels, causing ischemia and hypoxia in tumors and leading to tumor cell death [5]. Endostatin, one of the most potent antiangiogenic factors, was first isolated in Folkman's laboratory in 1997 and was reported to inhibit tumor blood vessel formation. Through continued studies by a Chinese scientist, recombinant human endostatin (rh-endostatin), an antiangiogenic drug, was approved by the State Food and Drug Administration (SFDA) of China for the treatment of non-small cell lung cancer (NSCLC), exhibiting synergistic efficacy with chemotherapy [6]. However, extensive research has not been conducted on normal vessel cells, which are deemed relatively mature and “gene-stable” cells and lack tumor features. Rh-endostatin can inhibit the proliferation, induce the apoptosis, and inhibit the migration of neovascular endothelial cells [7]. However, the role of rh-endostatin in the tumor microenvironment has yet to be elucidated.

Hypoxia is an important feature of solid tumors. However, an important role of antiangiogenic drugs is to block the blood supply of tumor cells, which can aggravate hypoxia in the tumor microenvironment. Several studies have shown that hypoxia not only promotes the invasion and metastasis of tumor cells but also plays an important role in resistance to angiogenesis [8, 9]. The improper (excessive) administration of antiangiogenic drugs can increase the migration and invasion of tumor cells, as verified by numerous studies [10, 11]. Following treatment with “improper (excessive)” antiangiogenic drugs, vascular endothelial cells, which are target cells involved in angiogenesis and important participants in tumor angiogenesis, undergo biological alterations as a result of dramatic changes in the microenvironment. We simulated the tumor microenvironment and hypoxic and hypoxic/starvation (H/S) microenvironments produced by improper antiangiogenic therapy in vitro and observed changes in the biological behavior of human umbilical vein endothelial cells (HUVECs) in these microenvironments.

## 2. Methods

### 2.1. Cell Culture, Hypoxia, and Hypoxia/Starvation

HUVECs (Peking Union Medical College Cell Bank, Beijing, China) were cultured in DMEM containing 10% FBS. Hypoxia (1% O_2_, 4% CO_2_, and 95% N_2_) was induced in a modular incubator (Billups–Rothenberg, San Diego, California). Hypoxia/starvation was induced by hypoxia and culture in serum-free DMEM.

### 2.2. MTT Assay for the Quantification of Cell Proliferation

MTT (3-(4,5-dimethylthiazol-2-yl)-2,5-diphenyl tetrazolium bromide) assays were used to measure mitochondrial function. HUVECs were seeded in 96-well plates (2000 cells/well) and incubated with different concentrations of rh-endostatin under different conditions. Then, 20 μl of MTT (Solarbio, 5 mg/ml, dissolved in PBS, pH 7.4) was added to the cells. After 4 h, the supernatant was removed, to which 150 μl of DMSO was added, and the mixture was gently shaken for 20 min. The absorbance was detected using a microplate reader (Bio-Rad Laboratories, Hercules, CA, USA) at a wavelength of 490 nm. Cell survival rate = (OD value of experimental group − OD value of blank control)/(OD value of control group − OD value of blank group) × 100% and cell inhibiting rate = 1 − cell survival rate.

### 2.3. Tube Formation Assays

HUVECs were seeded into a 48-well plate at a density of 4 × 10^4^ cells/well (400 μl of DMEM per well). The plate was precoated with 150 µl of Matrigel (BD Biosciences, Bradford, MA, USA). Place it on ice for 20 min to evenly distribute the matrix adhesive, then, place it in a 37°C incubator for 30 min to solidify the matrix adhesive. HUVECs were pretreated with different concentration rh-endostatin, then digest it into a single-cell suspension (final concentration: 5 × 10^5^/ml), 200 µl inoculated on the surface of the Matrigel. The HUVECs were then stained with calcein for 30 min at 37°C, after which the medium was replaced with DMEM. Images were captured with an inverted phase contrast microscope (Leica DMI6000B, 200× magnification). Tube-like structures were counted to evaluate tube formation ability. All the experiments were repeated three times independently.

### 2.4. Migration Assays

HUVECs (5 × 10^4^ cells/well) were seeded into transwell inserts (8 μm, Corning, Inc., Corning, NY, USA, without Matrigel) and add 600 µl DMEM containing 20% FBS as a chemotactic agent to the lower chamber. For the migration assay, the inserts were then placed in culture wells and incubated for 8 h. Then, the cells were rinsed and fixed in 4% paraformaldehyde for 10 min and then subjected to crystal violet (Beyotime, Haimen, Jiangsu, China) staining. Digital images (400x magnification) of the underside of the insert were captured with a microscope (ECLIPSE TS100, Nikon, Tokyo, Japan). The number of cells in six randomly selected fields per well per insert was counted.

### 2.5. Flow Cytometry (FCM)

HUVECs (5 × 10^5^) from each group were collected after the last treatment and resuspended in 50 μl of PBS. Next, the cells were incubated with 10 μl of FITC-conjugated antibodies against CD105 at a 1:100 dilution (BioLegend, Catalog Number: 323204) for 30 min at room temperature in the dark. Then, the cells were washed with binding buffer. The corresponding FITC-conjugated mouse immunoglobulin G1 isotype control (BioLegend, Catalog Number: 40011) was used as a control. Measurements were obtained with a FACSCanto II (BD Biosciences). Data analysis was performed using Flow Jo_V10 software (FlowJo LLC, Ashland, OR). The proportion of fluorescence positive cells (CD105) = CD105^+^ cells/total count of HUVECs.

### 2.6. Statistical Analysis

The statistical analysis was performed using SPSS 21.0 software. The measurement data are expressed as the mean ± SD of triplicate experiments. Statistical significance was determined by two-way ANOVA. The LSD test was applied for the comparison of multiple means with homogeneous variance, and Dunnett's T3 test was used for the comparison of multiple means with nonhomogeneous variance. A value of *p* < 0.05 was considered to indicate statistical significance.

## 3. Results

### 3.1. Rh-Endostatin Inhibits HUVEC Proliferation

**Figure 1:**
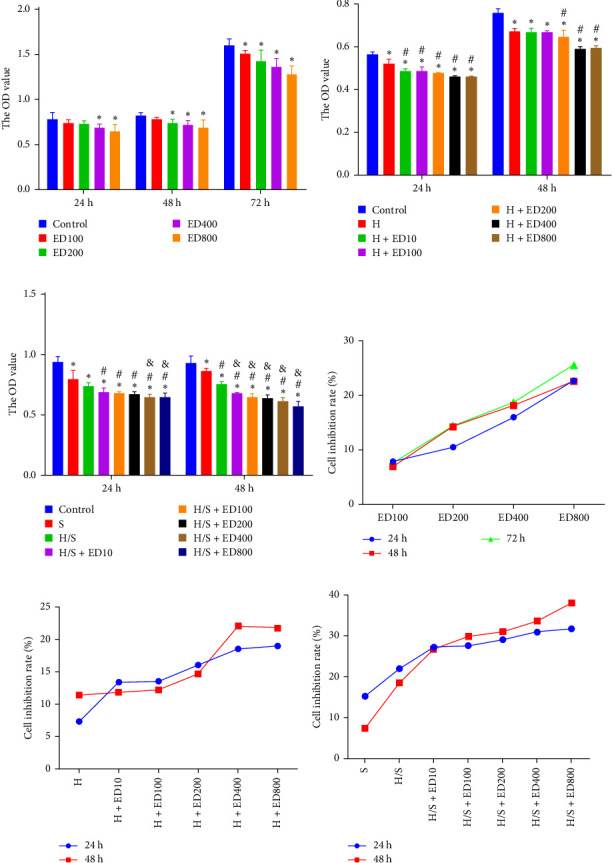
Rh-endostatin suppressed HUVEC proliferation under different conditions. (A–C) The MTT results for HUVECs following treatment with different concentrations of rh-endostatin under normoxic (control), hypoxic (H), and hypoxic/starvation (H/S) conditions, respectively, (D–F) the corresponding cell inhibition rates. ED100, ED200, ED400, and ED800 represent the concentrations of rh-endostatin (unit: μg/ml). (A) *⁣*^*∗*^*p* < 0.05, compared with the control; (B) *⁣*^*∗*^*p* < 0.05, compared with the control; ^#^*p* < 0.05, compared with H; (C) *⁣*^*∗*^*p* < 0.05, compared with the control; ^#^*p* < 0.05, compared with S; ^&^*p* < 0.05, compared with H/S.

Cell proliferation was assessed by the MTT assay. The cell inhibition rate was used to evaluate the effect of rh-endostatin on inhibiting HUVEC proliferation. HUVECs cultured under normoxic conditions were used as the control group. Compared with those in the control group, the proliferation of HUVECs cultured under hypoxic (H), starvation (S), and H/S conditions was inhibited (*p* < 0.05). Rh-endostatin inhibited the proliferation of HUVECs under normoxic, hypoxic, and H/S conditions, and this effect was dependent on dose; however, the time specificity was not significant (Figure 1). Based on the effects and characteristics of the different concentrations of rh-endostatin on the proliferation of HUVECs, in subsequent experiments, the concentrations of the various drugs used were 100, 200, 400, and 800 μg/ml under normoxic conditions and 10, 200, 400, and 800 μg/ml under hypoxic and H/S conditions.

### 3.2. Effects of Rh-Endostatin on HUVEC Tube-Like Structures

**Figure 2:**
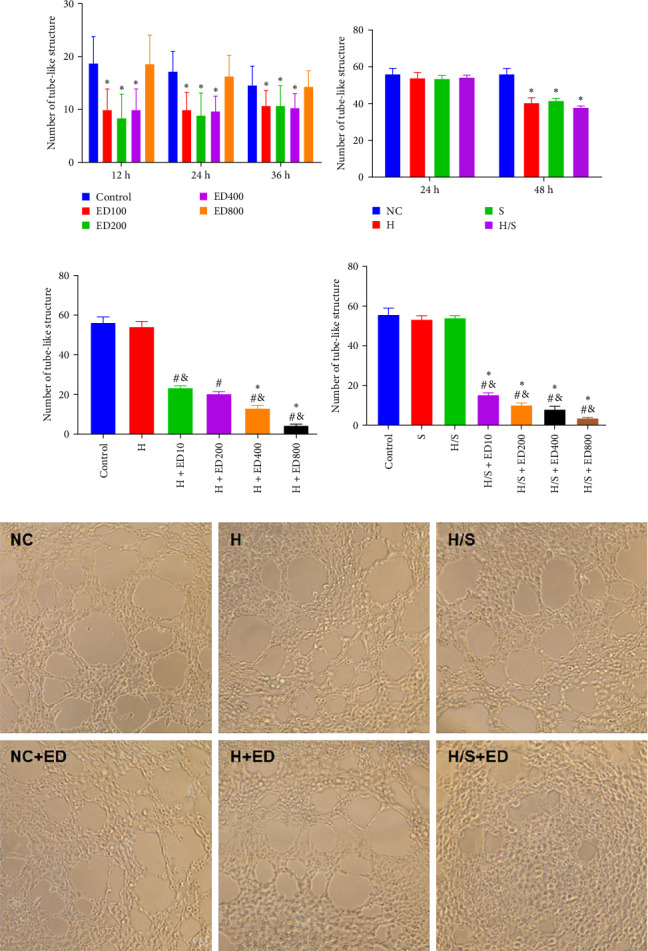
Effect of rh-endostatin on HUVEC tube-like formation under different conditions. (A) The number of tube-like structures after treatment with 10, 200, 400, and 800 μg/ml rh-endostatin for 12, 24, and 36 h under normoxic conditions (*⁣*^*∗*^*p* < 0.05, compared with the control). (B) The number of tube-like structures after exposure to hypoxic (H), starvation (S), and hypoxia/starvation (H/S) conditions for 24 and 48 h (NC: normoxic condition, *⁣*^*∗*^*p* < 0.05, compared with NC). (C) The number of tube-like structures after treatment with different concentrations of rh-endostatin for 24 h under hypoxic conditions (^#^*p* < 0.05, compared with the control; ^&^*p* < 0.05, compared with H; *⁣*^*∗*^*p* < 0.05, compared with H + ED10). (D) The number of tube-like structures after treatment with different concentrations of rh-endostatin for 24 h under hypoxic/starvation conditions (^#^*p* < 0.05, compared with the control; ^&^*p* < 0.05, compared with S; *⁣*^*∗*^*p* < 0.05, compared with H/S). (E) Images of tube formation under various conditions without/with rh-endostatin (200 μg/ml) for 48 h (original magnification ×20).

A tube formation assay was conducted to evaluate the tube formation ability of the HUVECs. When HUVECs under normoxic conditions were treated with rh-endostatin for 12, 24, and 36 h, there were fewer tube-like structures in the 100, 200, and 400 μg/ml rh-endostatin than in the control group; however, cell in the 800 μg/ml group were not affected. Cell tube formation did not significantly change in the hypoxia, starvation, or hypoxia/starvation groups after 24 h compared with that in the normoxia group; however, cell tube formation was inhibited after 48 h in cells incubated under hypoxic and starvation conditions. No significant differences were observed among the hypoxia group, starvation group, and hypoxia/starvation group. Treatment with rh-endostatin (10, 200, 400, or 800 μg/ml for 48 h) inhibited the tube formation of HUVECs under hypoxic and H/S conditions (Figure 2).

### 3.3. Effects of Rh-Endostatin on HUVEC Transwell Migration

**Figure 3:**
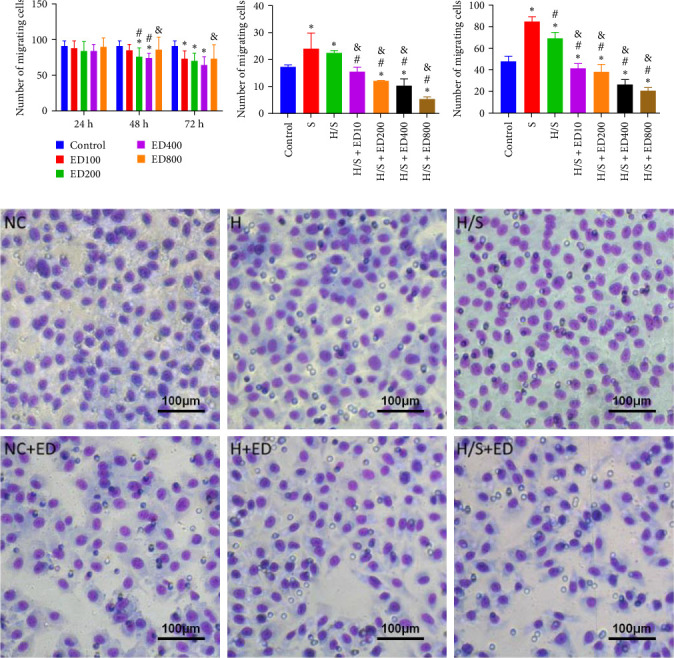
Transwell migration experiment. (A) The number of migrated cells after treatment with 100, 200, 400, and 800 μg/ml of rh-endostatin for 24, 48, and 72 h under normoxic conditions (control; *⁣*^*∗*^*p* < 0.05, compared with control). (B) The number of migrated cells after treatment with 10, 200, 400, and 800 μg/ml of rh-endostatin for 24 h under hypoxic conditions (H; *⁣*^*∗*^*p* < 0.05, compared with control; ^#^*p* < 0.05, compared with H). (C) The number of migrated cells after treatment with 10, 200, 400, and 800 μg/ml of rh-endostatin for 24 h under hypoxic/starvation (H/S) conditions (*⁣*^*∗*^*p* < 0.05, compared with control; ^#^*p* < 0.05, compared with S; ^&^*p* < 0.05, compared with H/S). (D) Transwell images under various conditions without or with rh-endostatin (200 μg/ml) for 24 h (magnification ×400).

The migration of HUVECs was evaluated with a transwell assay. Cell migratory ability was inhibited after treatment with rh-endostatin (200, 400, or 800 μg/ml for 48 h and 100, 200, 400, or 800 μg/ml for 72 h) under normoxic conditions. Compared with that of cells cultured under normoxic conditions, cell migration was enhanced after HUVECs were cultured under hypoxic conditions for 24 or 48 h. Rh-endostatin (10, 200, 400, and 800 μg/ml for 24 h) inhibited the migration of HUVECs under hypoxic and H/S conditions. However, there was no difference between the H + ED10 group and H + ED200 group or between the H/S + ED10 group and H/S + ED200 group (Figure 3).

### 3.4. FCM for Detecting the Expression of CD105

**Figure 4:**
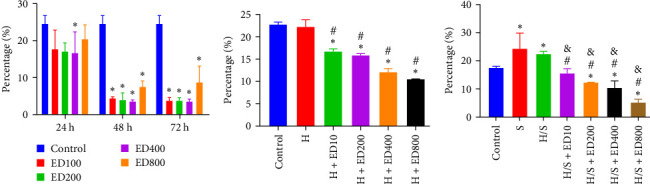
Effects of rh-endostatin on the HUVEC surface marker CD105. (A) The expression of CD105 after treatment with 100, 200, 400, and 800 μg/ml of rh-endostatin for 24, 48, and 72 h under normoxic conditions (control; *⁣*^*∗*^*p* < 0.05, compared with control). (B) The expression of CD105 after treatment with 100, 200, 400, and 800 μg/ml of rh-endostatin for 24 h under hypoxic conditions (H; ^#^*p* < 0.05, compared with control; *⁣*^*∗*^*p* < 0.05, compared with H). (C) The expression of CD105 after treatment with 100, 200, 400, and 800 μg/ml of rh-endostatin for 24 h under hypoxic/starvation conditions (*⁣*^*∗*^*p* < 0.05, compared with control; ^#^*p* < 0.05, compared with S; ^&^*p* < 0.05, compared with H/S).

Rh-endostatin significantly downregulated CD105 expression after 24 (400 μg/ml), 48, and 72 h (100, 200, 400, and 800 μg/ml). Compared with those in the normoxia group, the expression of CD105 was not different in the hypoxia (24 h) group, but starvation and hypoxia/starvation upregulated the expression of CD105. Rh-endostatin (10, 200, 400, and 800 μg/ml for 24 h) downregulated the expression of CD105 under hypoxic conditions. There was no significant difference in the expression of CD105 between the H + ED10 group and H + ED200 group or between the H + ED400 group and H + ED800 group. Under H/S conditions, compared with starvation or H/S conditions, rh-endostatin (10, 200, 400, or 800 μg/ml for 24 h) downregulated the expression of CD105. There was no difference between the H/S+ED10 group and control group or between the H/S + ED10 group and H/S + ED200 group (Figure 4).

## 4. Discussion

It has previously been demonstrated that interrupting antiangiogenic therapy greatly increases the invasiveness of recurrent and regenerated tumor cells [12]. Tumor cells proliferate quickly and tumor blood vessels are abnormal, that is, twisted, tangled, and highly permeable; thus, it is impossible to provide adequate oxygen and nutrients, which will inevitably lead to hypoxia in the tumor microenvironment. In the tumor microenvironment, antiangiogenic agents may aggravate hypoxia, and hypoxia can activate signaling pathways in tumor cells and increase tumor cell tolerance to adverse conditions, promote the formation of blood vessels, and lead to tumor invasion and metastasis [13]. In the process of inhibiting angiogenesis, endothelial cells are the target cells of antiangiogenic drugs due to their presence in tumors and the hypoxic microenvironment [14]. The addition of antiangiogenic drugs results in a change in the microenvironment during treatment [15]; however, whether the biological behavior of endothelial cells changes due to severe changes in the microenvironment remains unknown. Our previous work revealed that hypoxia + high-dose bevacizumab promotes the migration and tube formation ability of endothelial cells, indicating that antiangiogenic therapy exacerbates hypoxia and increases the malignancy of tumor cells; moreover, hypoxia + high-dose bevacizumab promotes the malignant transformation of endothelial cells [16]. Therefore, we sought to investigate whether this occurrence is typical of all antiangiogenic therapies. Currently, research on this topic is extremely limited. Herein, we designed an experiment to observe the effect of a representative antiangiogenic drug (rh-endostatin) on the biological behavior of HUVECs under hypoxic and H/S conditions and to explore the underlying mechanism involved.

The migration and angiogenesis of endothelial cells are important processes and complex biological phenomena. These processes can be influenced by vascular endothelial growth factor (VEGF), basic fibroblast growth factor (bFGF), CXC chemokines, and other angiogenic factors [17, 18]. These factors affect matrix metalloproteinases (MMPs), which degrade the extracellular matrix and cause endothelial cells to move away from the confines of the matrix. Then, endothelial cells migrate to surrounding tissues and gradually form mature reticular vessels [19]. These findings indicate that the balanced network involved in angiogenesis is complicated. Importantly, the long-term results of inhibiting one of the factors or one of the pathways associated with angiogenesis are poor. Therefore, the combined use of different single-target or multitarget drugs may be a judicious strategy [20]. The results of the present study suggested that compared with that of the control group, the biological behavior (proliferation, tubular structure formation, and migration) of HUVECs was inhibited after treatment with rh-endostatin, but the inhibitory effect was relatively weak. Our findings showed that under hypoxic conditions, the pan-target drug rh-endostatin does not promote the malignant biological behavior of HUVECs, but bevacizumab does. In a H/S environment, rh-endostatin suppressed the biological behavior of HUVECs. As a pan-target drug, does rh-endostatin suppress HUVECs? Herein, we found that rh-endostatin inhibits HUVEC proliferation in vitro in a time-dependent and dose-dependent manner. Similarly, HUVEC migration and tube formation were suppressed by rh-endostatin under hypoxic and normoxic/starvation conditions.

Additionally, under normoxic, hypoxic, and normoxic/starvation conditions, HUVECs treated with rh-endostatin exhibited downregulated expression of CD105. CD105 is known as an endothelial factor and is expressed on the membranes of highly proliferative endothelial cells. It plays an important role in many pathological processes and conditions, including angiogenesis, inflammation, and tumors [21–23]. As a specific marker of the vascular endothelium, CD105 is not expressed or expressed at a very low level in vessels of normal tissues, but is highly expressed in vasculogenic or tumor tissues [24]. The proliferation of vascular endothelial cells is related to tumor angiogenesis status; therefore, CD105 can be used as a marker of tumor microvessel density and has shown potential in studies of markers that can be used to evaluate the prognosis of patients with tumors [22]. Our previous experimental results showed that at a high concentration, bevacizumab upregulates the expression of CD105 on HUVECs under hypoxia [16], but the results of the present study showed that rh-endostatin downregulates the expression of CD105 on HUVECs, which suggests that rh-endostatin has an antiangiogenic effect though suppressing the biological behavior of HUVECs.

In vitro simulation of hypoxia, starvation, and hypoxia/starvation in tumor tissue can induce malignant biological behaviors in HUVECs to a certain extent [25]. The effects of antiangiogenic drugs on HUVECs under hypoxic conditions are different from those under normoxic conditions. The malignant biological behaviors of HUVECs were promoted by high-dose bevacizumab under hypoxic conditions; in contrast, under hypoxic and H/S conditions, rh-endostatin inhibited the malignant biological behavior of HUVECs induced by hypoxia. The results suggested that for endothelial cells in a hypoxic environment, compared with bevacizumab, rh-endostatin inhibits the malignant biological behavior of HUVECs. Hypoxia and hypoxia/starvation promote the malignant biological behavior of HUVECs, but these malignant biological behaviors are inhibited by rh-endostatin, in addition to the downregulation of CD105 expression. Herein, we suggest that CD105 can reflect the malignant biological behavior of HUVECs. The measurement of CD105 may be a useful tool for monitoring tumor angiogenesis and evaluating the efficacy of antiangiogenic therapies.

Overall, the biological behavior of HUVECs is an important part of angiogenesis. Herein, we conducted experiments to evaluate the function of the multitarget drug re-endostatin on HUVECs in vitro and found that rh-endostatin can suppress HUVECs under hypoxic and H/S conditions.

## Data Availability

The data used to support the findings of this study are available from the corresponding author upon request.
